# The arachidonic acid metabolite 11β-ProstaglandinF2α controls intestinal epithelial healing: deficiency in patients with Crohn’s disease

**DOI:** 10.1038/srep25203

**Published:** 2016-05-03

**Authors:** Sabrina Coquenlorge, Laurianne Van Landeghem, Julie Jaulin, Nicolas Cenac, Nathalie Vergnolle, Emilie Duchalais, Michel Neunlist, Malvyne Rolli-Derkinderen

**Affiliations:** 1 INSERM, UMR913, Nantes, F-44093, France; 2 Université Nantes, Nantes, F-44093, France; 3 Institut des Maladies de l’Appareil Digestif, CHU Nantes, Hôpital Hôtel-Dieu, Nantes, F-44093, France; 4 Centre de Recherche en Nutrition Humaine, Nantes, F-44093, France; 5 Centre de Pathophysiologie, CHU Purpan, Toulouse, France; 6 INSERM UMR-1043 CNRS UMR-5282, Toulouse, France

## Abstract

In healthy gut enteric glial cells (EGC) are essential to intestinal epithelial barrier (IEB) functions. In Crohn’s Disease (CD), both EGC phenotype and IEB functions are altered, but putative involvement of EGC in CD pathogenesis remains unknown and study of human EGC are lacking. EGC isolated from CD and control patients showed similar expression of glial markers and EGC-derived soluble factors (IL6, TGF-β, proEGF, GSH) but CD EGC failed to increase IEB resistance and healing. Lipid profiling showed that CD EGC produced decreased amounts of 15-HETE, 18-HEPE, 15dPGJ_2_ and 11βPGF_2_α as compared to healthy EGC. They also had reduced expression of the L-PGDS and AKR1C3 enzymes. Produced by healthy EGC, the 11βPGF_2_ activated PPARγ receptor of intestinal epithelial cells to induce cell spreading and IEB wound repair. In addition to this novel healing mechanism our data show that CD EGC presented impaired ability to promote IEB functions through defect in L-PGDS-AKR1C3-11βPGF_2_α dependent pathway.

Compelling evidence has demonstrated that defects in mucosal healing are central to Crohn’s Disease (CD) pathogenesis and prognosis. In particular several studies have reported that intestinal mucosal lesions precede inflammation and are considered as a predictive factor of relapse^1^,^2^,^3^. Mucosal healing has further been suggested to represent a treatment goal and a predictive factor for sustained clinical remission in CD^4^,^5^,^6^.

The intestinal epithelium is a dynamic interface between the environment and the organism that must constantly preserve its integrity to maintain digestive and barrier functions. After injury, mucosal repair is a key process to restore epithelium lining and functions such as permeability control leading ultimately to intestinal homeostasis. Three main concomitant regenerative processes participate to mucosal healing and include epithelial restitution, that involves cell spreading and migration into the wound, cell proliferation and differentiation^7^. It has now been well demonstrated that IEB functions, including intestinal healing, are regulated by neighboring cells, the so-called microenvironment, and in particular the enteric nervous system (ENS)^8^.

The ENS is an integrative neuronal network localized along the gastrointestinal tract that regulates key digestive functions such as gut motility and mucosal secretion^9^,^10^. ENS is composed of enteric neurons and enteric glial cells (EGC) that outnumber enteric neurons by a factor of 4 to 10^11^ and share common markers and functional properties with central nervous system astrocytes^12^,^13^,^14^,^15^,^16^. EGC form a dense network that surrounds intestinal crypts, and are located at less than 2 μm from intestinal epithelial cells (IECs). A large number of studies from our group and others have now well demonstrated that EGC are key regulators of IEB homeostasis and functions^17^,^18^. In particular EGC impact IEC major functions *via* paracrine signaling. For instance they inhibit IEC proliferation *via* the release of transforming growth factor-β (TGF-β) and 15-deoxy-delta12,14-Prostaglandin J2 (15dPGJ_2_), a derivative of n-6 (omega-6) polyunsaturated fatty acids (PUFA), and activation of Peroxisome proliferator-activated receptorγ (PPARγ)-dependent pathways^19^,^20^. Importantly, EGC promote mucosal healing *via* enhanced cell spreading/restitution and pro-epidermal growth factor (proEGF) secretion^21^ and decrease intestinal permeability *via* S-nitrosoglutathione (GSNO) production^22^.

Of major interest, EGC ablation in transgenic murine models leads to histopathological alterations reminiscent to CD^23^,^24^,^25^. Some human studies have reported abnormalities of the EGC network in CD patients with mostly altered expression of EGC markers such as S100β and GFAP^25^,^26^,^27^. However whether CD EGC have altered ability to regulate IEC functions remains largelly unknown. A very recent study has shown that EGC of CD patients have reduced ability to regulate paracellular permeability^28^ but their impact on the control of IEB repair remains currently unknonwn.

In this study, we hypothesized that CD EGC will show impaired functional phenotype as compared to ‘healthy’ EGC, thus participating to CD-associated defects in IEB mucosal healing. Using a non-contact co-culture model of human EGC isolated from CD or control patients and IEC, we assessed whether CD EGC have differential impact on IEC sealing, spreading and healing than control EGC, and we identified glial-soluble factors and signaling pathways involved.

## Results

### CD EGC express the same level of glial markers than control EGC

**Figure 1:**
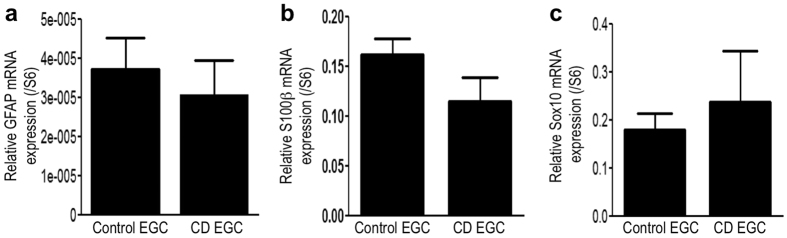
Human enteric glial cells characterization: control and CD EGC express the same level of glial markers. (**a**) *GFAP*, (**b**) *S100β*, (**c**) *Sox10* mRNA expressions were measured by RT-qPCR and expressed as a ratio to *S6* mRNA. n = 19 control EGC and n = 17 CD EGC; Mann-Whitney test.

Previous studies have demonstrated that during IBD, the expression of glial markers, GFAP and S100β were altered. A decrease of GFAP expression was observed in non-inflamed area of CD patients compared to control patients, whereas an increase of GFAP was observed in inflamed area of ulcerative colitis and CD patients^25^,^26^,^27^. To characterize our EGC primary cultures, we measured *GFAP* (Fig. 1a), *S100β* (Fig. 1b) and *Sox10* (Fig. 1c) mRNA expression by RT-qPCR and observed no significant differences between control and CD EGC.

### Primary cultures of EGC from CD patients have lost their ability to speed up IEB healing

**Figure 2:**
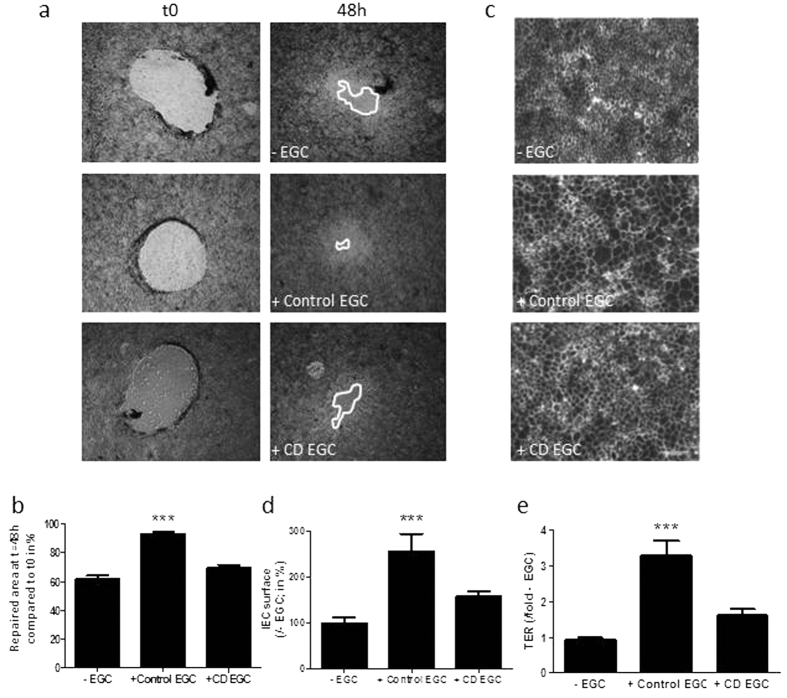
Functional impact of human enteric glial cells (EGC) on IEB healing. IEC properties were evaluated in Caco-2 monolayers after three days of indirect co-culture with control (+Control EGC) or CD EGC (+CD EGC) or without co-culture (−EGC). (**a**) Representative pictures of epithelial healing of IEC monolayers after three days of indirect co-culture with control (+Control EGC), CD EGC (+CD EGC) or without co-culture (−EGC). (**b**) Epithelial healing was measured as a percentage of restitution between t0 and 48 h. n = 7 −EGC and CD EGC; n = 12 control EGC; Kruskal-Wallis test; ***p < 0.005 as compared to IEC without EGC. (**c**) Representative ZO-1 immunostaining of IEC monolayers after three days of indirect co-culture with control (+Control EGC), CD EGC (+CD EGC) or without co-culture (−EGC) (Scale bar = 100μm). (**d**) Epithelial spreading is the measure of cell area delimited by ZO-1 staining and expressed as percentage of IEC surface measured in (−EGC) condition, and (**e**) the transepithelial electrical resistance (TER) was measured and compared to TER of IEC –EGC considered as 1 (n = 20 −EGC, n = 14 control EGC; n = 11 CD EGC; Kruskal-Wallis test; ***p < 0.005 as compared to −EGC.

In previous studies we have shown that EGC stimulate intestinal epithelial cell monolayer healing in part through spreading acceleration. These effects are associated with an increase in Caco-2 cell transepithelial electrical resistance (TER), indicative of increased sealing of the monolayer^21^. To determine whether CD EGC have impaired functional impact on IEB, we compared effects of EGC isolated from CD patients *vs.* control patients on Caco-2 cell TER, spreading and healing in a non-contact co-culture model. Using an *in vitro* mechanically induced wound-healing model, we showed that while control EGC enhanced wound closure, CD EGC had no significant impact (Fig. 2a,b). Consistent with previous work^29^, human control EGC significantly increased Caco-2 cell spreading and monolayer resistance (Fig. 2c–e). In contrast, CD EGC did not impact spreading (Fig. 2c,d) or TER (Fig. 2e) as compared to Caco-2 monolayer cultured alone. These results demonstrate that EGC from CD patients have impaired effects on IEB healing.

### CD EGC express the same level of IL-6, TGF-β1, EGF and GCLc than control EGC

**Figure 3:**
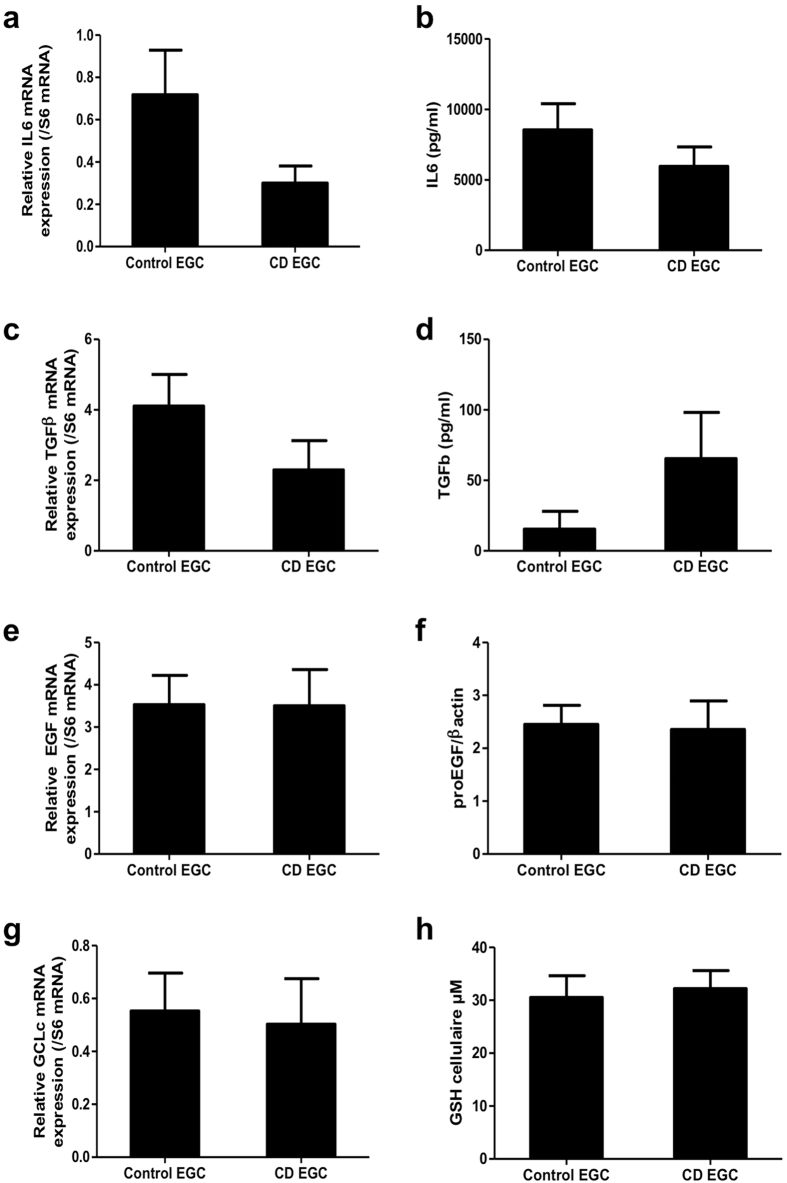
No difference in EGC-derived soluble factors expression between control and CD EGC. Study of the expression of four glial mediators (IL6, TGF-β1, EGF and glutathione GSH) was performed by ELISA (**b,d,h**), Western-Blot (**f**) or qPCR of the mediator or of its producing enzyme as GCLc (glutamate-cysteine ligase, catalytic subunit) (**a,c,e,g**). n = 11–12 control EGC and n = 5–10 CD EGC; Mann-Whitney test.

We next hypothesized that CD-associated impairment of EGC functions was due to altered soluble factor production. Thus, we first quantified four EGC-derived soluble factors known to regulate IEB functions. Those included Interleukin 6 (IL6), TGF-β1, EGF and glutathione. Control EGC and CD EGC expressed similar levels of *IL6* (Fig. 3a) and *TGF-β1* (Fig. 3c) mRNA (p = 0.21 and p = 0.06 respectively), and released similar concentration of IL6 (Fig. 3b) and TGF-β1 (Fig. 3d) (p = 0.74 and p = 0.48 respectively). *EGF* (Fig. 3e) and *glutamate cysteine ligase catalytic subunit* (*GCLc*; Fig. 3g) mRNA were expressed at similar levels in control and CD EGC. Consistently proEGF (Fig. 3f) and glutathione (Fig. 3h) proteins were produced at equivalent levels by control and CD EGC.

### CD EGC produce less 15-HETE, 18-HEPE, 15dPGJ_2_ and 11βPGF_2_α

Previous work from our group and others has shown that EGC secrete PUFA metabolites that could regulate IEB functions such as 15dPGJ_2_, 15-HETE and PGE_2_^19^,^28^,^30^. We next sought to determine whether PUFA metabolite release was altered in CD EGC *vs.* control EGC. Mass spectrometry data on EGC conditioned medium show that among 31 PUFA metabolites assessed, 21 were detected in both CD and control EGC conditioned medium (Table 1). The 15dPGJ_2_, 9-alpha, 11-beta prostaglandin F2 (11βPGF_2_α), 15-hydroxyeicosatetraenoic acid (15-HETE) and 18-hydroxyeicosapentaenoic acid (18-HEPE) concentrations were significantly reduced in CD EGC conditioned medium compared to control EGC conditioned medium (Table 1).

### CD EGC express less L-PGDS and AKR1C3 than control EGC

**Figure 4:**
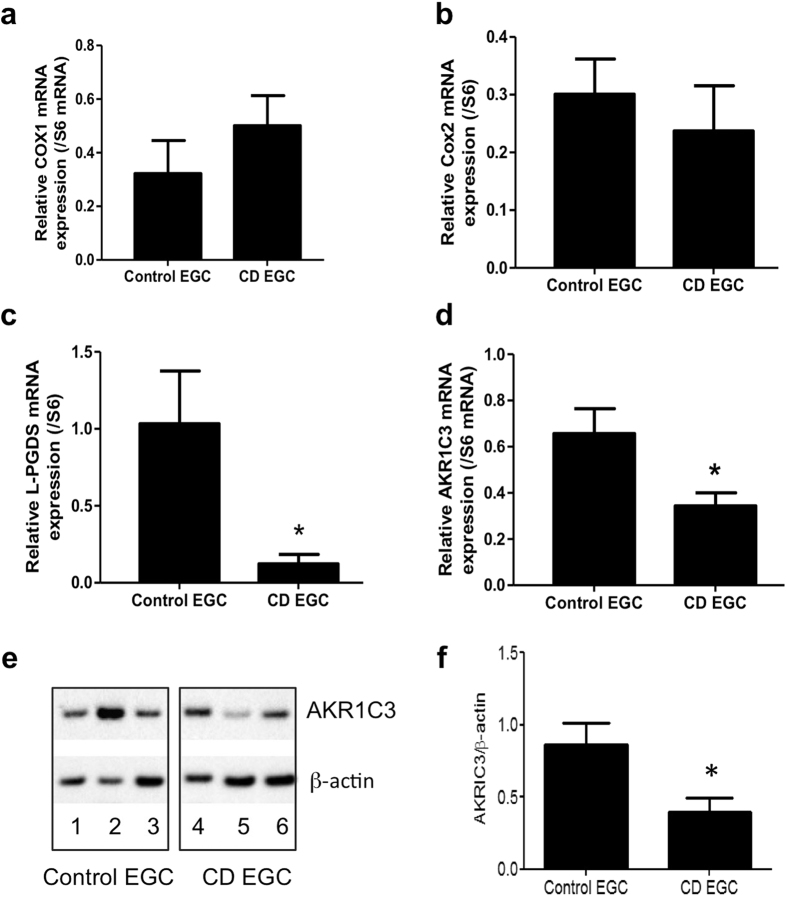
CD EGC present a defect in L-PGDS and AKR1C3 expressions. Quantitative PCR analyze of *Cox1, Cox2*, *L-PGDS* and *AKR1C3* mRNA expression related to *S6* mRNA expression. Control and CD EGC expressed the same level of *Cox1* and *Cox2* (a and b) mRNA expression but CD EGC had a significantly reduced expression of *L-PGDS* (**c**) and *AKR1C3* (**d**); n = 1–13 control EGC and n = 11–12 CD EGC. (**e**) Representative western blotting analyzes of AKR1C3 expression in Control EGC (patient 1, 2, 3) and CD-EGC (patients 4, 5, 6). (**f**) Quantification of AKR1C3/β-actin from n = 11 control EGC and n = 8 CD EGC. Data represent mean ± SEM,; Mann-Whitney test; *p < 0.05 as compared to control EGC.

Both 15dPGJ_2_ and 11βPGF_2_α derive from transformation of PGD_2_, which is synthetized by lipocalin like prostaglandin D synthase (L-PGDS) and cyclooxygenases (COX) enzymes. The aldo-keto reductase family 1 member C3 (AKR1C3) converts PGD_2_ to 11βPGF_2_α. To assess if this pathway was altered in CD EGC, we measured *Cox-1, Cox-2* and *L-PGDS* but also *AKR1C3* mRNA expression. In CD EGC, while *Cox-1* (Fig. 4a) and *Cox-2* (Fig. 4b) mRNA levels were unchanged, *L-PGDS* (Fig. 4c) and *AKR1C3* (Fig. 4d) mRNA expressions were significantly reduced in CD EGC as compared to control EGC. We further demonstrated that the protein expression of AKR1C3 was significantly reduced in CD-EGC as compared to control EGC (Fig. 4e,f). These results show for the first time that human EGC expressed the enzyme AKR1C3 but especially indicate that L-PGDS and AKR1C3 pathway is down regulated in EGC derived from CD patients that are thereby defective for 11βPGF_2_α production.

### 11βPGF_2_α reproduces control EGC impact on IEC healing and spreading

**Figure 5:**
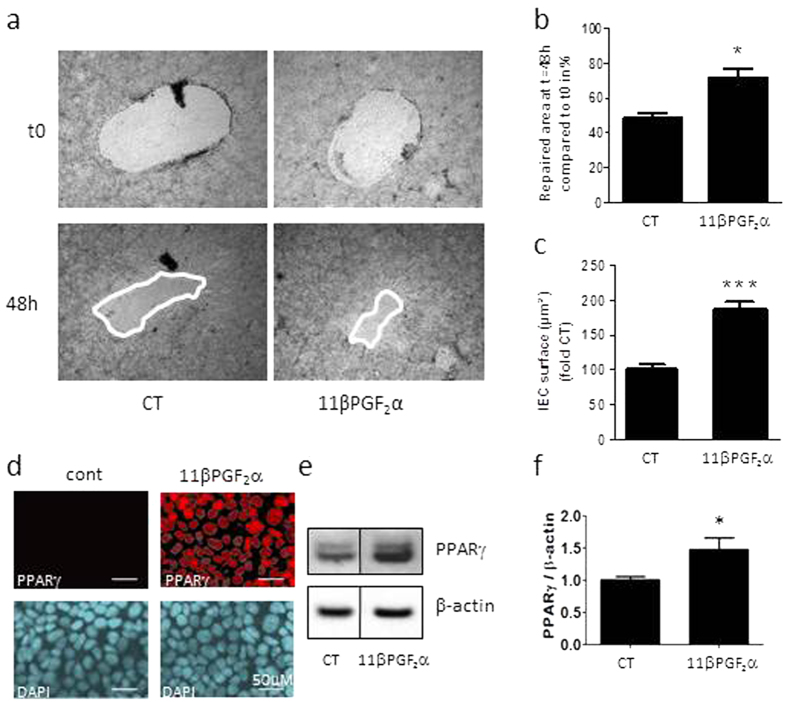
Functional impact of 11βPGF_2_α on IEB healing. IEC properties were evaluated in Caco-2 monolayers after three days of culture in presence of lipid mediator 11βPGF_2_α or not (CT).(**a**) Representative pictures of epithelial healing of IEC monolayers cultured without treatment (CT) or with 11βPGF_2_α for two days. (**b**) Quantification of epithelial healing was calculated as a percentage of healing between t0 and 48 h. n = 5–12; Kruskal-Wallis test; *p < 0.05 as compared to IEC without treatment. (**c**) Epithelial spreading was expressed as percentage of CT IEC surface. n = 5–8 independent experiments performed in duplicates; Kruskal-Wallis test; ***p < 0.005 as compared to CT. (**d**) IEC properties were also evaluated in Caco-2 monolayers after two days of co-culture with Control EGC or CD EGC in presence of the lipid mediator 11βPGF_2_α or not (−). Quantification of epithelial healing was calculated as a percentage of healing between t0 and 48 h. n = 3; Kruskal-Wallis test; *p < 0.05 as compared to CD EGC-Caco2 co-cultures without treatment. ^§^p < 0.05 as compared to Control EGC-Caco2 co-cultures without treatment. (**e**) PPARγ immunostaining on Caco-2 treated 5min with 11βPGF_2_α compared to ZO-1 staining. n = 3 independent experiments, scale 20 μM. (**f**) Western blotting analyzes of PPARγ expression in Caco-2 treated 24 hours with 11βPGF_2_α. (**g**) Quantification of PPARγ/β-actin related to the average control ratio taken as 1. Data represent mean ± SEM, n = 4 independent experiments; Kruskal-Wallis test; *p < 0.05 as compared to cont.

The impact of glial 15dPGJ_2_ on IEC was already described^19^, therefore we focused on 11βPGF_2_α effects on IEB wound closure. IEB healing was significantly accelerated by 11βPGF_2_α (Fig. 5a,b), and IEC spreading was also strongly enhanced by 11βPGF_2_α (Fig. 5c). To assess whether 11βPGF_2_α could restore CD EGC function, we have added 11βPGF_2_α in the culture medium of EGC co-cultivated with Caco-2 monolayer.11βPGF_2_α supplementation in CD EGC – Caco-2 co-cultures significantly increased IEB healing but has no additional effect when added in control EGC – Caco-2 co-cultures (Fig. 5d). These data show that the functional defect presented by CD EGC could be fixed by 11βPGF_2_α addition.

As it has been proposed that AKR1C3 or the prostaglandin D2 and its derivatives may regulate ligand access to the orphan nuclear receptor PPARγ^31^, we have analyzed whether 11βPGF_2_α could activate PPARγ. Immunocytochemistry showed a transient increase of PPARγ nuclear localization after 5min of IEC treatment with 11βPGF_2_α (Fig. 5e). PPARγ expression was also significantly increased after 24 hours of IEC treatment with 11βPGF_2_α (Fig. 5f,g). These data demonstrate that 11βPGF_2_α reproduced control EGC effects on IEB, namely IEC spreading and IEB healing promotion, and activate the orphan nuclear receptor PPARγ.

### PPARγ agonist induces IEC spreading and IEB healing

**Figure 6:**
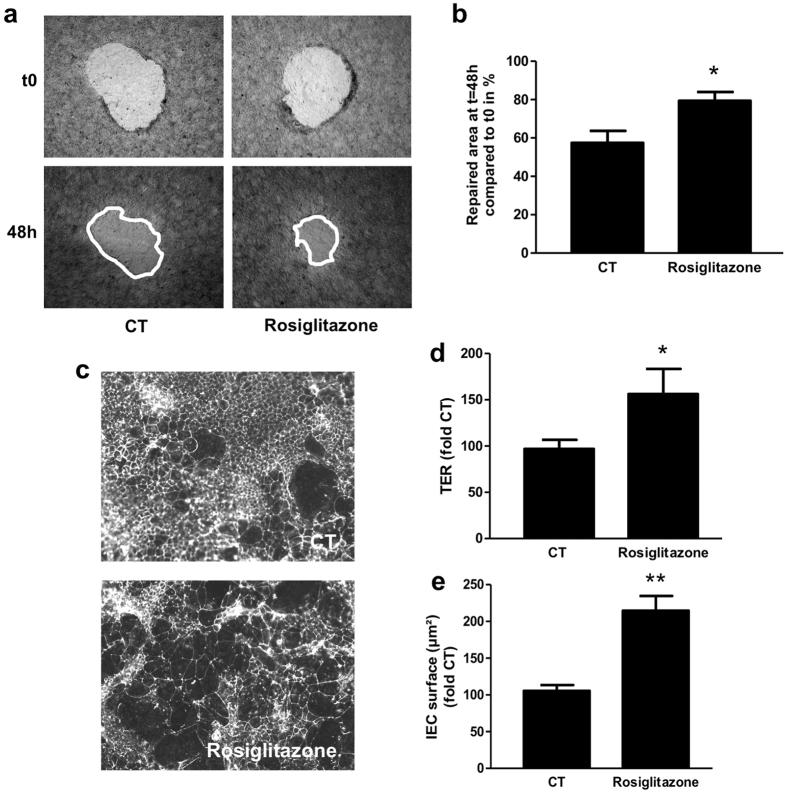
PPARγ agonist reproduces control EGC effect on IEB healing. IEC properties were evaluated in Caco-2 monolayers after three days of treatment with the PPARγ agonist rosiglitazone. (**a**) Representative pictures of epithelial healing of IEC monolayers cultured without treatment (CT) or with rosiglitazone. (**b**) Quantification of epithelial healing was calculated as a percentage of healing between t0 and 48 h. n = 6 independent experiments (**c**) Representative ZO-1 immunostaining of IEC filters treated or not with rosiglitazone, (**d**) Transepithelial electrical resistance (TER) expressed as percentage of CT TER and (**e**) epithelial spreading expressed as percentage of CT IEC surface. n = 5 independent experiments; Mann-Whitney test; *p < 0.05; **p < 0.01 as compared to CT.

We then assessed the impact of PPARγ agonist on IEB healing and TER, and on IEC spreading. The PPARγ agonist, rosiglitazone, resulted in an accelerated healing (Fig. 6a,b) and an increased cell spreading (Fig. 6c,e) and TER (Fig. 6d). These results show that as 11βPGF_2_α, PPARγ agonist enhances IEC spreading and IEB healing.

### Functional defect of CD EGC on IEB properties involved DP2 and PPARγ-dependent pathways

**Figure 7:**
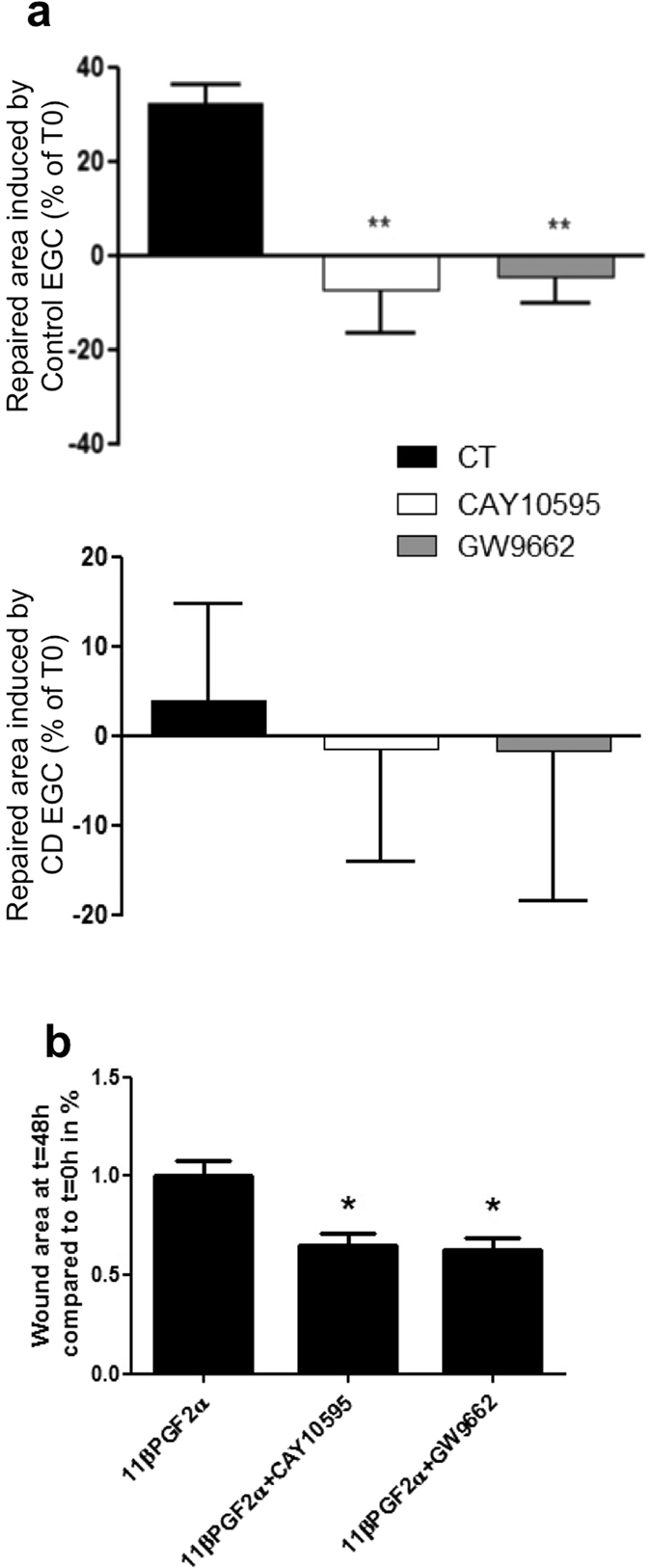
Implication of DP receptors and PPARγ in IEB healing induced by EGC. DP (CAY10595) and PPARγ (GW9662) antagonists were applied directly on IEC cultivated alone, on IEC co-cultivated with Control EGC or on IEC co-cultivated with CD EGC during three days. Quantification of epithelial healing was calculated as the difference between the percentage of healing between t0 and 48 h for co-cultures and the percentage of healing between t0 and 48 h for Caco-2 cultivated alone (**b**) CAY10595 and GW996 were also added to IEC culture in presence of 11βPGF_2_α. Quantification of epithelial healing was calculated as a percentage of healing between t0 and 48 h and compared to 11βPGF_2_α healing considered as one. n = 4–7 independent experiments; Kruskal-Wallis test; *p < 0.05; **p < 0.01 as compared to without treatment.

To assess if 11βPGF_2_α is the mediator of EGC regulation of IEB, and to unravel how it does, we blocked 11βPGF_2_α dependent pathways in our co-culture model of IEB and control or CD EGC. As 11βPGF_2_α can act through PPARγ but also through FP or DP2 receptor, we used the selective FP antagonist (AL8810), DP2 antagonist (CAY10595) as well as PPARγ antagonist (GW9662). CAY10595 and GW9662 significantly blocked IEB healing induced by control EGC but had no effect on IEB healing when Caco-2 monolayer was cultivated in the presence of CD EGC (Fig. 7a). The AL8810 had no significant effect upon wound healing in any condition tested (not shown). Both CAY10595 and GW9662 also blocked the increase of IEB healing induced by 11βPGF_2_α (Fig. 7b). These data show that DP2 and PPARγ-dependent pathways are responsible for IEB healing induced by 11βPGF_2_α glial production, and that CD EGC lack these regulations.

## Discussion

**Figure 8:**
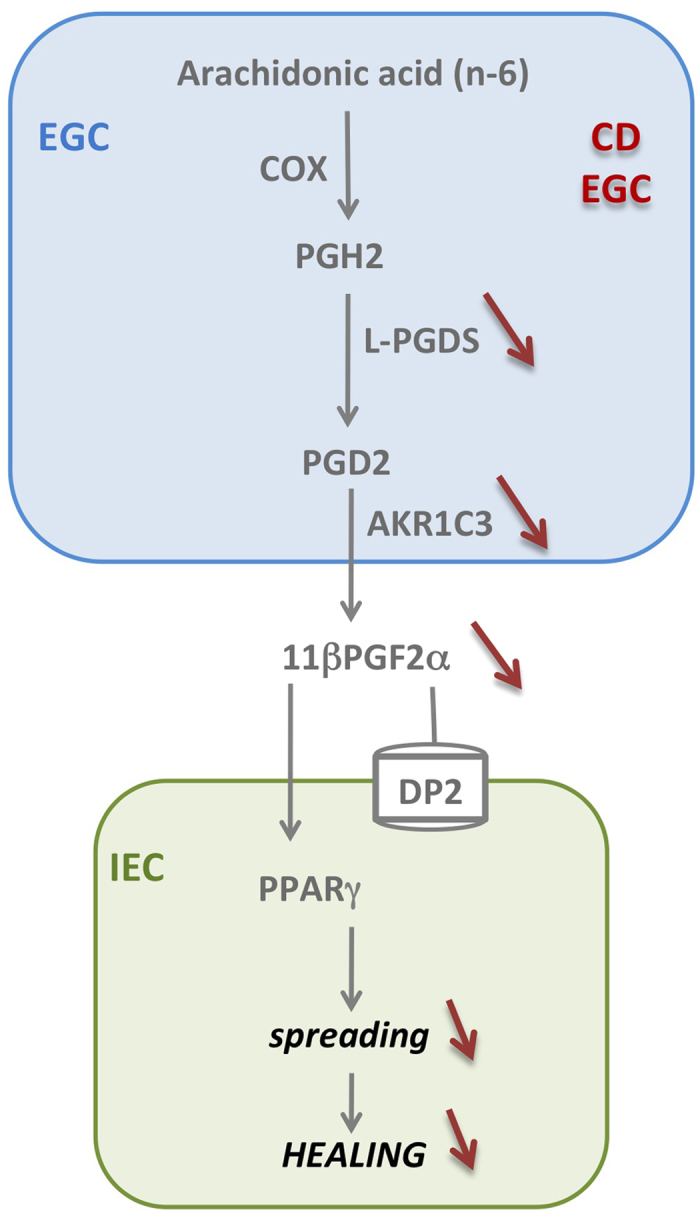
Schematic representation of the control of IEB healing by human EGC from control and CD EGC. Human EGC can produce PUFA metabolites derived from n-3 or n-6. Among the 21 mediators secreted by human EGC, only two by-products of prostaglandin D2 (PGD_2_), 15dPGJ_2_ and 11βPGF_2_α were misproduced by CD EGC that express less L-PGDS and AKR1C3 but the same level of COX1 and COX2. 15dPGJ_2_ and 11βPGF_2_α glial production regulates IEB healing through PPARg and DP2 dependent pathway.

The main goal of this work was to assess whether primary cultures of EGC from CD patients have altered impact on IEB healing, compared to EGC from control patients. A second goal of this study was to better characterize human EGC lipidic secretome and to study its possible impact on IEB repair. Our data unravel that human EGC from control patients produce the prostaglandin 11βPGF_2_α that induces IEC spreading and accelerates IEB wound healing. We demonstrate that 11βPGF_2_α acts through DP2- and PPARγ-dependent pathways. In addition we demonstrate that in contrast to control EGC, CD EGC do not enhance TER, spreading or wound closure, indicating that CD EGC have lost their ability to accelerate sealing and healing. We show that this defect is due to a down-regulation of L-PGDS and AKR1C3 expression and a reduced production of 11βPGF_2_α (Fig. 8).

One of the main findings of this work is that CD EGC did not accelerate sealing of epithelial monolayer, did not stimulate cell spreading and as a result, did not promote wound closure, as opposed to their healthy homologues. These data strongly implies that defects in mucosal healing observed in CD could be, at least in part, due to EGC that have lost their abilities to promote cell restitution. Altogether our *ex vivo* functional studies indicate that primary cultures of EGC isolated from CD patients exhibit loss of function as compared to EGC isolated from control patients. These findings suggest that CD EGC have altered intrinsic functional phenotype. Regarding the recent work of Pochard et *al*., that shows that CD EGC have decreased properties to control IEB permeability^28^, it is thus tempting to speculate that impaired intrinsic functions of EGC from CD patients predispose and/or directly participate to the disease onset or favor relapse.

Another important result of this work is the description of human EGC lipidic signature. In previous studies we have already described the rat EGC lipidic signature^28^ and have shown that 15dPGJ2 was produced by rat EGC^19^, and the prostaglandin PGE_2_ has already been shown to be produced by enteric glia^32^ as well as by central glial cells^33^,^34^,^35^,^36^. In the present study, among 31 PUFA metabolites measured in human EGC conditioned medium, we detected 19 other PUFA derivatives, in addition to these two prostaglandins previously described. These derivatives are produced by the activation of three major AA metabolic pathways, *i.e*. COX; LOX and p450 signaling pathways, which are active in EGC. According to the low stability of PUFA catabolic products, we cannot rule out that the PUFA derivatives that we did not detect are not produced in EGC. Concerning the role of these metabolites, little is known about their specific regulation of the neuro-glio-epithelial unit. We have already shown that glial production of 15dPGJ_2_ exerts anti-proliferative and pro-differentiating effects on IEC^19^. Beside its impact on IEC, 15dPGJ_2_ can regulate other cells of the glial microenvironment. For instance, 15dPGJ_2_ is a neuroprotective agent^37^,^38^,^39^ and is believed to be responsible for neutrophil recruitment^40^. On another hand, the prostaglandin PGD_2_ and the COX pathway have been described to regulate enteric nervous system excitability^41^,^42^, and more specifically glial production of PGE_2_ has been shown to potentiate neuronal response to bradykinin^32^. In this study we concentrated on determining the role of 11βPGF_2_α, the primary plasma metabolite of PGD_2_ *in vivo*^43^. Up to now 11βPGF_2_α had no specific intestinal function. It inhibits ADP- or thrombin-induced human platelet aggregation^44^, induces human bronchial smooth muscle contractions^45^, inhibits adipocyte differentiation^46^ and promotes prostate cell proliferation^47^. To the best of our knowledge this is the first study that demonstrates that 11βPGF_2_α is produced in the intestine by EGC and that it massively increases IEC spreading, enhances cell restitution and thereby wound closure.

Described as equipotent to PGF_2_α, 11βPGF_2_α is believed to act through FP receptors, although this still needs to be clearly demonstrated. We did not observed significant effect of FP antagonist on IEB healing but have demonstrated that 11βPGF_2_α, as well as EGC, induced healing through type 2 PGD_2_ receptor (DP2)- and peroxysome-proliferator activated receptor (PPARγ)-dependent pathways (Fig. 7). Besides canonical pathways such as Wnt/β-catenin or Notch, recent data have shown that PPARγ signaling is also a key pathway in the control of IEC functions^48^. For instance, PPARγ activation inhibits IEC proliferation and promotes cell differentiation^49^,^50^. In the present study we demonstrate that PPARγ activation, induced by EGC or not, can promote IEC spreading and IEB wound closure. The barrier-promoting impact of the 11βPGF_2_α-PPARγ-pathway that we have observed in the intestine, could also be of interest in other epithelial tissues, and could explains protective effects of this pathway against lethal influenza infection with lung viral load reduction for example^51^.

In addition we have compared human EGC lipidic secretome from control *versus* CD patients. Among the 21 PUFA metabolites measured in control EGC conditioned media, four of them are significantly less present in CD EGC conditioned media: the 15-HETE, the 18-HEPE, the 15dPGJ2 and the 11βPGF_2_α. In this study we have demonstrated that 11β-PGF_2_α promotes wound healing. As mentioned above, 15dPGJ2 has neuroprotective effects^37^,^38^,^39^ and is already described to regulate IEC proliferation and differentiation^19^. 15-HETE regulates different vascular functions^52^,^53^,^54^,^55^,^56^,^57^ and concerning effects on epithelium, 15-HETE has been shown to induce cell growth of pre-confluent non-differentiated intestinal epithelial cells^58^ and to increase Caco-2 TER and decrease permeability^59^. We have very recently demonstrated that the glial production of 15-HETE is responsible for the control of ZO-1 expression and IEB permeability, and that this regulation is lost in CD^28^. Very little is known about 18-HEPE role, but one paper suggests that 18-HEPE could play a role in mucosal repair^60^. Thus, it is likely that CD EGC deficit in 15-HETE, 18-HEPE, 15dPGJ2 and 11βPGF_2_α production overall participates to abnormalities/alterations observed during CD^1^,^2^,^3^,^6^,^26^,^61^,^62^,^63^.

Finally, our study suggest that 11βPGF_2_α reduced production by CD EGC results from reduced L-PGDS and AKR1C3 expression in CD-EGC as compared to control EGC. Indeed 15dPGJ_2_ and 11βPGF_2_α derive from prostaglandin D2 (PGD2), which results from prostaglandin H2 (PGH2) isomerization by prostaglandin D synthase (PGDS). While IEC, mast cells and fibroblasts express H-PGDS, the most representative form of PGDS in the gut^64^,^65^ EGC express the lipocalin-PGDS (L-PGDS)^19^ We have shown that EGC also express the aldo-keto reductase AKR1C3 that converts PGD2 to 11β-PGF_2_α. First identified as an enzyme involved in steroid metabolism, AKR1C3 could metabolize a broad spectrum of carbonyl compounds and has broad role in the development of tumor diseases^66^. We could speculate that the role of glial AKRIC3 is wider than the production of 11β-PGF_2_α and the consequent induction of IEC spreading. The function and regulation of AKRIC3, as the ones of 15-HETE and 18-HEPE in EGC deserve further investigation.

Collectively our results demonstrate that EGC from CD patients exhibit loss of function as compared to control EGC, due to defects in L-PGDS, AKR1C3 and 11βPGF_2_α expression/synthesis. Thus this work confirms and extends the emerging major role of EGC on intestinal homeostasis and mucosal repair after injury^17^,^18^,^67^. Enteric glia not only regulates epithelial cells, but also forms a cellular and molecular bridge between enteric nerves^68^,^69^, enteroendocrine cells^70^,^71^ and immune cells^72^ that lets suspect even broader impact of EGC upon intestinal functions. Our results not only imply that CD EGC could be actors of CD pathogenesis or development, but also establish a molecular basis for developing and testing therapeutic strategies by targeting 11βPGF_2_α production or DP2-PPARγ dependent pathways.

## Methods

### Enteric glial cells

Cultures of human enteric glial cells (EGC) were obtained according to the procedure described by Soret *et al.*^29^. Briefly, human EGC originated from intestinal resections of control patients (patients having undergone surgery for colorectal cancer, 10cm from the tumor area) and from patients with an established diagnosis of CD according to international criteria. All intestinal resections were macroscopically healthy. 15 control (age 56– 89 y; sex ratio 5men:10women) and 11 CD (age, 17–63 y; sex ratio 4men:7women) patients were included in this study. Patients gave their informed consent to take part in the study and all procedures were performed according to the guidelines of the French Ethics Committee for Research on Human and registered under the no. DC-2008–402. An immunocytochemistry study was performed to verify the purity of the EGC population as described by Soret *et al.*^29^. EGC cultures presenting more than 80% of GFAP-, Sox10-, and S100*β* positive cells were used for these experiments, at passage 3.

### Co-culture model and spreading, TER and healing measurement

The human IEC line Caco-2 was cultured in DMEM medium (Gibco^®^, Life Technologies, Carlsbad, CA, USA). For spreading and resistance experiments, Caco-2 cells and EGC were respectively seeded on 24-well transwell filters (Pore size 0.45 μm, Corning, Avon, France) at 90 × 10^3^ cells/cm^2^ and in 24-well plates at 7,9 × 10^3^ cells/cm^2^ for three days. The IEC size was measured thanks to anti-zonula occludens-1 immunostaining (see Immunofluorescence staining). The transepithelial electrical resistance (TER) was measured every day with an epithelial volt-ohmmeter (EVOM, World Precision Instruments, Inc). For wound healing experiments, Caco-2 cells and EGC were respectively seeded onto 6-well filters at 122 × 10^3^ cells/cm^2^ and in 6-well plates at 2,1 × 10^3^ cells/cm^2^ for 15 days. Caco-2 monolayers were wounded with the 0.5–10 μl tip of a pipette. Each hole was photographed at 0 and 48 h by using a microscope (Axiovert 200M; Zeiss; objective lens 5×) with Axiovision software and surface was quantified with ImageJ software (National Institute of Health, Bethesda, Maryland, United States). Epithelial healing was calculated as the percentage of the healing after two days of treatments or EGC co-culture compared to t0.

### Immunofluorescence staining and western blotting

IEC transwell filters were fixed in PBS 4% paraformaldehyde for 30 min. The IEC size was measured thanks to anti-zonula-occludens-1 (ZO-1) immunostaining. Briefly, fixed filters were incubated for 30 min at RT with PBS containing 0.5% Triton X-100 (Sigma-Aldrich, Saint-Louis, MO, USA) and 10% horse serum (Merk Millipore, Billerica, MA, USA; PBS-TX-HS) and then incubated with a mouse monoclonal antibody anti-ZO-1 diluted in PBS-TX-HS (1:500; Life Technologies) for 2 h at RT. After washing with PBS, filters were incubated with an anti-mouse CY-3 (1:500; Jackson ImmunoResearch, West Grove, PA, USA) for 45 min at RT. After washing with PBS containing DAPI (Sigma-Aldrich) for the first wash, filters were mounted on slides for fluorescence microscopy analysis. Images were acquired with a digital camera (Olympus DP 50) coupled to a fluorescence microscope (Olympus IX 50). Cell surface area was measured with ImageJ software. An average of 149.9+/−8.6 IEC was analyzed for each experimental condition. PPAR-γ nuclear localisation was studied by the same immunostaining procedure using anti- PPAR-γ antibody (sc-7273 diluted 1/500). The same antibody diluted 1/500 was used for western blotting procedure.

### ELISA

The concentrations of IL-6 (BD OptEIA^TM^ ELISA Set, BD Biosciences, Franklin Lakes, NJ, USA), TGF-β1 (Cytoset, Novex^®^, Life Technologies) and GSH (glutathione assay kit, Sigma-Aldrich) were determined in EGC culture supernatants by ELISA according to the manufacturer’s instructions. Absorbance measurements were performed at 450 nm on a spectrophotometric enzyme-linked immunosorbent sandwich assay (ELISA) plate reader (Varioskan^®^, Thermo Scientific, Rockford, IL, USA) using the SkanIt software (Thermo scientific).

### PUFA dosage

The PUFA dosage was performed as described Le Faouder *et al.*^73^. This innovative method has been improved for the simultaneously measurement of 31 lipids derived from the n-3 and n-6 PUFA that are 6-keto-prostaglandin F1α (6kPGF_1_α), thromboxan B_2_ (TXB_2_), prostaglandin E_2_ (PGE_2_), prostaglandin E_3_ (PGE_3_), prostaglandin A_1_ (PGA_1_), 8-iso prostaglandin A_2_ (8-isoPGA_2_), 15-Deoxy-Delta12,14-Prostaglandin J_2_ (15d-PGJ_2_), 9-alpha,11-beta prostaglandin F_2_ (9α,11βPGF_2_ or 11βPGF_2α_), lipoxin A_4_ (LxA_4_), resolvin D_1_ (RvD_1_), leukotrien B_4_ (LTB_4_), leukotrien B_5_ (LTB_5_), 10(S), 17(S)-protectin (PDx), 18-hydroxyeicosapentaenoic acid (18-HEPE), 15-hydroxyeicosatetraenoic acid (15-HETE) and 12-HETE, 8-HETE, 5-HETE, 17-hydroxy-docosahexaenoic acid (17-HDoHE) and 14-HDoHE, 14,15-epoxyeicosatrienoic acid (14,15-EET) and 11,12-EET, 8,9-EET, 5,6- EET, 5-oxoeicosatetraenoic acid (5-oxo-ETE). Briefly, the 24 lipids of interest and 3 deuterated internal standards (LxA_4_-d5, LTB_4_-d4, 5-HETE-d8), were separated by LC-MS/MS analysis on HPLC system (Agilent LC1290 Infinity) coupled to Agilent 6460 triple quadrupole MS (Agilent Technologies) equipped with electro-spray ionization operating in negative mode. Reverse-phase HPLC was performed using ZorBAX SB-C18 column (2.1 mm; 50 mm; 1.8 μm) (Agilent Technologies) with a gradient elution. Mobile phase A consisted of water, ACN and FA (75/25/0.1); Solvent B: ACN, FA (100/0.1). Compounds were separated with a linear gradient to 85% B from 0 to 8.5 min and 100% B to 9 min. Isocratic elution continued for 1 min at 100% B then 100% A was reached at 11 min and maintained to 12 min. The flow rate was 0.35 mL/min. The autosampler was set at 5 °C and the injection volume was 5 μL. Data were acquired in MRM mode with optimized conditions (fragmentors and collision energy). Peak detection, integration and quantitative analysis were done using Mass Hunter Quantitative analysis software (Agilent Technologies). PUFA profile was established in EGC conditioned media. 75 000 HOG were plated in T25 flasks and placed in 5 ml defined DMEM media supplemented with 100 IU/ml penicillin, 100 μg/ml streptomycin and 10% heat-inactivated fetal calf serum. After 3 days, conditioned media were centrifuged at 14,000 rpm for 5 minutes at 4 °C and 500 μl frozen at −80 °C until analysis.

### Real-time quantitative PCR analysis

IEC grown on filters were lyzed in RA1 buffer (Macherey-Nagel, Düren, Germany) in order to study mRNA and protein expression. According to the manufacturer’s recommendations, total RNA extraction from cells was performed with Nucleospin RNAII kit (Macherey-Nagel) and 1 μg purified RNA was denatured and processed for reverse transcription using Superscript II reverse transcriptase (Invitrogen). PCR amplifications were performed using the Absolute Blue SYBR green fluorescein kit (Roche) and run on a Rotor-Gene (Qiagen, Venlo, The Netherlands). The following primers (Sigma-Aldrich) were used:

**AKR1C3 (Aldo-keto reductase family 1, member C3)** # NM_003739.5

Forward Primer: 5′-AGTAAAGCTTTGGAGGTCACA-3′

Reverse Primer: 5′-ACTCTGGTCGATGAAAAGTGG-3′

**GCLc (Glutamate cysteine ligase, catalytic subunit)** # NM_001197115.1

Forward Primer: 5′-AGGTGACATTCCAAGCCTGC-3′

Reverse Primer: 5′-CCCCAGCGACAATCAATGTC-3′

**Prostaglandine D2 synthase (L-PGDS or PTGDS) #** NM_000954.5

Forward Primer: 5′-AGAAGAAGGCGGCGTTGTCC-3′

Reverse Primer: 5′-CCACCACTGACACGGAGTAGG-3′

**Prostaglandin-endoperoxide synthase1 (prostaglandinG/H synthase and cyclooxygenase) (PTGS1 or COX1)** NM_

Forward Primer: 5′-TCCATGTTGGTGGACTATGG-3′

Reverse Primer: 5′-GTGGTGGTCCATGTTCCTG-3′

**Prostaglandin-endoperoxide synthase2 (prostaglandinG/H synthase and cyclooxygenase) (PTGS2 or COX2)** NM_000963.3

Forward Primer: 5′-TGGGAAGCCTTCTCTAACCTC-3′

Reverse Primer: 5′-TCAGGAAGCTGCTTTTTACCTT-3′

**IL6** # NM_000600

Forward Primer: 5′-CAATGAGGAGACTTGCCTGGTGAA -3′

Reverse Primer: 5′-TGTGGTTGGGTCAGGGGTGGTT-3′

**TGF-β 1** #NM_000660

Forward Primer: 5′-GTCACCGGAGTTGTGCGGCA-3

Reverse Primer: 5′-CTCGGCGGCCGGTAGTGAAC-3

**Ribosomal protein S6 (S6)** # NM_001010

Forward Primer: 5′-CCAAGCTTATTCAGCGTCTTGTTACTCC-3′

Reverse Primer: 5′-CCCTCGAGTCCTTCATTCTCTTGGC-3′

Relative quantification of gene expression was determined using the standard curve method and endogenous control ribosomal protein S6 mRNA.

### Immunoblotting

Lysates from RA1 extraction were precipitated and pellets were resuspended in PSB/TCEP (Macherey-Nagel). Samples were processed for electrophoresis using NuPAGE MES SDS buffer kit (Life technologies) and separated on 4–12% Bis-Tris gels (NuPAGE, Life Technologies). Proteins were transferred to nitrocellulose membranes with the iBlot™ system (Life Technologies). After blocking with TBS/0.1% Tween20/5% nonfat dry milk for 1 hour, blots were incubated overnight at 4 °C with primary antibodies diluted in TBS/5% nonfat dry milk for rabbit anti-proEGF (1:500; Pierce, Thermo Scientific), and mouse anti-β-actin (1:10000; Sigma-Aldrich). Immunoblots were probed with the appropriate horseradish peroxidase-conjugated secondary antibodies (Life Technologies) and visualized by chemiluminescence (ECL, Biorad, Hercules, CA, USA). Quantitative analysis was performed using ImageJ software. The value of protein immunoreactivity was normalized to the amount of β-actin immunoreactivity.

### Treatment of IEC monolayers

IEC cultivated on filters as described in our *“Co-culture model”* section were also treated with 11βPGF_2_α (5 μM; Cayman Chemical, Ann Arbor, MI, USA) or the PPARγ agonist (Rosiglitazone; 5 μM; Sigma-Aldrich) for three days, and spreading, TER, or healing were measured as described above. IEC co-cultivated with control or CD EGC were also treated with FP receptor antagonist (AL8810; 1 μM; Cayman Chemical), DP2 receptor antagonist (CAY10595; 10 nM; Cayman Chemical), or PPARγ antagonist (GW9662; 10 μM; Sigma-Aldrich) for two days, and healing was measured as described above.

### Statistics

Data are expressed as the mean ± SEM of three to ten independent experiments. The significance of differences was determined using a Mann-Whitney test to compare two groups and a one-way analysis of variance (ANOVA), Kruskal-Wallis test followed by a Dunn’s post-test, to compare three groups or more. Differences were considered statistically significant for p < 0.05.

### Study approval

Patients gave their informed consent to take part in the study and all procedures were performed according to the guidelines of the French Ethics Committee for Research on Human. All experimental protocols were approved by local Committee on Ethics and Human Research and the Inserm (Institut national de la santé et de la recherche médicale) and registered under the no. DC-2008-402.

## Additional Information

**How to cite this article**: Coquenlorge, S. *et al.* The arachidonic acid metabolite 11β-ProstaglandinF2α controls intestinal epithelial healing: deficiency in patients with Crohn's disease. *Sci. Rep.* **6**, 25203; doi: 10.1038/srep25203 (2016).

## Figures and Tables

**Table 1:** Human enteric glial cells secrete PUFA metabolites.

	6kPGF1α	TxB2	PGE3	11β-PGF2α	PGF2α	PGE2	PGD2	
control	165.19	674.25	573.32	**386.46**	326.92	252.31	103.01	(pg/ml)
	138.45	619.46	658.93	198.97	316.03	222.67	60.68	SD
CD	871.97	525.72	584.30	*204.97	172.87	1181.72	130.69	(pg/ml)
	1676.83	290.22	655.02	110.81	109.30	1618.89	80.08	SD
	LxA4	8isoPGA2	PGA1	7MaR1	PD1	LTB4	**18-HEPE**	
control	827.89	625.24	6.87	652.02	206.51	79.84	**7176.48**	(pg/ml)
	520.19	735.16	24.76	951.28	252.52	108.10	8170.11	SD
CD	803.29	1067.13	25.83	21.26	65.11	70.86	*315.25	(pg/ml)
	459.53	1086.41	44.59	39.40	107.30	81.08	140.46	SD
	**15dPGJ2**	**15-HETE**	17-HDoHE	14-HDoHE	8-HETE	12-HETE	5-HETE	
control	**535.19**	**2009.81**	3664.70	4727.55	1099.49	3939.48	309.10	(pg/ml)
	488.10	1662.99	3444.67	3413.61	987.09	2414.31	231.79	SD
CD	*46.73	*800.78	1789.32	3377.03	594.34	2894.82	300.91	(pg/ml)
	21.39	385.62	3163.79	2918.26	910.33	1675.66	493.48	SD
non detected		LxB4	RvD2	RvD1	LTB5			
5,6-DiHETE	14,15-EET	5oxoETE	11,12-EET	8,9-EET	5,6-EET			

31 polyunsaturated fatty acid (PUFA) metabolites were quantified by liquid Chromatography/tandem Mass Spectrometry in EGC supernatants. Among the 21 mediators secreted by human EGC, the 15-HETE, the 18-HEPE and two by-products of prostaglandin D_2_, the 15dPGJ_2_ and the 11βPGF_2_α were less present in CD EGC conditioned media as compared to control EGC conditioned media. n = 14 control EGC and n = 11 CD EGC; Mann-Whitney test; *p < 0.05 as compared to control EGC.
